# Role of neuroticism and perceived stress on quality of life among patients with dry eye disease

**DOI:** 10.1038/s41598-022-11271-z

**Published:** 2022-04-30

**Authors:** Napaporn Tananuvat, Sasiwimon Tansanguan, Nahathai Wongpakaran, Tinakon Wongpakaran

**Affiliations:** 1 Department of Ophthalmology, Faculty of Medicine, Chiang Mai University, Chiang Mai, Thailand; 2 Department of Psychiatry, Faculty of Medicine, Chiang Mai University, 110 Intawaroros Rd., T. Sriphum, A. Muang, Chiang Mai, 50200 Thailand

**Keywords:** Human behaviour, Quality of life

## Abstract

This hospital-based, cross-sectional observational study aimed to examine whether neuroticism has an impact on stress that is related to dry eye disease (DED) and quality of life (QOL). One hundred participants who had DED completed the Dry Eye-Related Quality-of-Life Score (DEQS) questionnaire, a 5-level EQ-5D (EQ-5D-5L), Neuroticism Inventory (NI), and 10-Item Perceived Stress Scale (PSS). Hierarchical linear regression was applied to determine the predictive effect of the independent variables. Participants’ mean age was 50.91 ± 14.3 years, and females totalled 89.0%. Hierarchical linear regression analysis showed that DESQ-Ocular symptoms were the strongest predictor for QOL either assessed by DEQS or EQ-5D, and its effect was lessened when perceived stress and neuroticism were added to the model. The final model explained up to 30–39% variance of the QOL, compared with 13–32% by DESQ-Ocular symptoms alone. QOL of the patients with DED, is not only related to eye symptoms but perceived stress. Moreover, neuroticism was a strong predictor contributing to the QOL among patients with DED. The study showed a significant association between perceived stress, neuroticism and the QOL of patients with DED. Personality has some impact on both subjective dry eye symptoms and impact on daily life, along with the general health-related QOL.

## Introduction

Dry eye disease (DED), one of the most frequent eye problems with global prevalence ranging from 5 to 50%, represents an important public health problem^1^. Its most common, consistent risk factors include aging, female, Asian ethnicity, meibomian gland dysfunction, contact lens wear, and computer use^1^. Symptoms of ocular discomfort and pain associated with DED can have negative effects on physical and psychological functions, while the impaired visual performance may impose restrictions in daily activities such as reading, driving, and using a computer or smartphone devices. Thus, DED can impact the overall individual quality of life (QOL) and reduce work productivity, through its adverse effect on visual performance^2–6^.

According to the 2017 International Dry Eye Workshop (DEWS) report, DED is defined as “a multifactorial disorder of the ocular surface characterized by a loss of homeostasis of the tear film, and accompanied by ocular symptoms, including tear instability and hyperosmolarity, ocular surface inflammation and damage, and neurosensory abnormalities”^7^. Despite more understanding of the pathophysiology of DED, management remains complex and challenging due to the chronicity, incurability as well as poor correlation between symptoms and signs^8,9^. In addition, treatments are mostly palliative and can remain lifelong leading to economic burden from both direct and indirect costs^1,10,11^.

DED can adversely affect patients’ QOL. An extensive review revealed that patient-reported symptoms of DED generally improved after treating with topical formulations for tear replacement, tear stimulation or anti-inflammatory therapy compared with baseline or a control treatment^12^. However, as satisfaction and QOL are evaluated by the patients’ subjective experience, other psychological factors related to emotion should come into play. A study with a substantial sample in Korea demonstrated a relationship between anxiety/depression and QOL among patients with DED^5^. Similarly, another study denoted a relationship with depression^13,14^, and even suicidal ideation. Such psychological distress is certainly related to the QOL.

Individuals reporting a high level of perceived stress are more likely to have dry eye symptoms. DED was significantly associated with stress in both young^15^ and older populations^16^. In addition, perceived stress, anxiety sensitivity, and a fear of anxiety-related sensations^17^ predicts the intensity of dry eye symptoms above and beyond depressive and anxiety symptoms^18^. Like perceived stress, anxiety sensitivity constitutes a domain-specific appraisal^19^.

Perceived stress and anxiety sensitivity are also closely related to neuroticism, a personality trait that has long been studied and found to be one of the major contributors to psychological distress. Neuroticism is related to perceived stress, depression^20,21^, anxiety sensitivity and QOL, either physical or emotional domains^22–26^. Growing evidence is emerging regarding the relationship between neuroticism, psychological distress and symptoms of DED. Individuals with high neuroticism tend to experience negative emotions, such as anxiety and anger, leading to susceptibility to psychological distress and vulnerability to stress^27^. It comprises a common trait that has been studied among patients with DED. Most studies have suggested the significance between neuroticism and dry eye symptom assessed by the DEQS^28–30^. However, one study noted that no association between the severity of signs or symptoms of DED and neuroticism was observed^31^.

Concerning the QOL of patients with DED, well-established evidence indicates a strongly significant relationship between dry eye symptoms and impact on daily life. As mentioned, other psychological factors could also contribute to either dry eye symptoms or QOL. Little is known how perceived stress and neuroticism play a role in the QOL of patients with DED. This study aimed to examine how psychological factors, i.e., perceived stress, and the personality trait of neuroticism impacted the relationship between dry eye symptoms and QOL, assessed either by a disease-specific measurement using the DEQS or by general measurement using the EQ-5D. We hypothesized that in addition to dry eye symptoms, perceived stress and neuroticism would demonstrate some effect on the QOL in this population.

## Materials and methods

This prospective cross-sectional study was approved by the Institute Review Board before being initiated (study code: OPT-2561-005562) and followed the Declaration of Helsinki. All volunteers signed written informed consent forms after a complete explanation.

### Sample size estimation

According to the number of predictors between two to four, and a sample size calculator on https://www.statskingdom.com/sample_size_regression.html, a minimum sample size of 67 was required for a significant level of 0.05, power of 0.8, and medium effect size.

### Study participants

This study recruited 100 participants at the Department of Ophthalmology, Chiang Mai University Hospital between 2018 and 2019. The eligible criteria included adult subjects aged ≥ 18, participating on a voluntary basis. The participants with a history of surgery within the past 6 months prior to the study were excluded.

The diagnostic criteria for DED complied with those defined by DEWS^32^ including (1) presence of ocular symptoms using the Ocular Surface Disease Index score ≥ 13, and (2) presence of one of the dry eye homeostasis markers; tear film break-up time (TBUT) ≤ 5 s or positive corneal fluorescein staining (CFS). Participants were excluded if they had the best-corrected visual acuity (BCVA) worse than 6/18, ocular surface inflammation or infection, ocular surgery within six months, systemic diseases or disabilities affecting daily life activities including psychological disorders.

All participants underwent complete ophthalmic examination for both eyes, including measuring BCVA and intraocular pressure. Additional dry eye tests including corneal fluorescein staining (CSF), tear film break-up time (TBUT), and Schirmer’s test were provided. CSF scores were assigned on a scale of 0 to 3 based on the van Bijsterveld grading system^33^ and TBUT was measured using fluorescein staining without anesthesia. Participants were asked to blink several times. The interval between the last complete blink and the first dry spot on the cornea was measured three times, and the average value was used for statistical analysis. Schirmer’s test was performed with anesthesia. After drying the excess tears, the Schirmer strip was placed at the lateral one third of the lower fornix for 5 min. The strip was then removed, and the wetting length of the filter paper was measured in mm.

### Procedure

All 100 participants were asked to complete the DEQS-TH questionnaire, health-related QOL questionnaire, i.e., the 5-level EQ-5D (EQ-5D-5L), perceived stress scale, and neuroticism inventory.

### Instruments

#### The Dry Eye-related Quality-of-Life Score (DEQS)

The DEQS questionnaire contains 15 questions addressing two subscales: *Bothersome Ocular Symptoms* (six items) and *Impact on Daily Life* (nine items). Each question was evaluated for frequency and severity, based on a 5-point scale ranging from “none of the time” (0) to “all of the time” (4). Higher scores indicated more severe symptoms and poorer QOL. The Thai version of the Dry Eye-related QOL Score (DEQS-Th) is a valid and reliable measurement. In this study, Cronbach’s alpha was 0.82 and 0.92 for *Bothersome Ocular Symptoms* and *Impact on Daily Life*, respectively. Factor analysis of the *Impact on Daily Life* subscale yielded the highest factor loadings on the impact on home, work or study and worsened when watching (0.846), whereas the lowest was found on depression (0.659).

#### EuroQol‑5‑Dimensions 5‑Level (EQ‑5D‑5L)

The EQ-5D, developed by EuroQoL, is composed of five items concerning ‘mobility’, ‘self-care’, ‘usual activities’, ‘pain/discomfort’ and ‘anxiety/depression’^34^. Respondents are asked to rate on a 5-point Likert scale ranging from 1—‘no problems’, 2—‘slight problems’, 3—‘moderate problems’, 4—‘severe problems’, and 5—‘unable to/extreme problems’. The score ranges from 0 to 1, with 0 meaning death and 1 meaning complete health. However, the index score can also have a negative value, meaning worse than dead. The Thai version EQ-5D-5L was validated and the index score was used in this study^35^. In this study, Cronbach’s alpha was 0.740. Factor analysis yielded the factor loadings on self-care and anxiety/depression the most (0.781 and 0.758, respectively), whereas on mobility the least (0.619).

#### 10-item perceived stress scale (PSS-10)

The PSS-10 is a questionnaire to evaluate to what extent individuals feel stress in the past month. It comprises a 10-item self-report using a 5-point Likert scale format (0 = never to 4 = very often^36^. The total score ranges from 0 to 40. Higher scores indicate greater perceived stress. The Thai version demonstrated good reliability and validity^37^. In this study, Cronbach’s alpha was 0.803.

#### 15-item Neuroticism inventory (NI-15)

The NI-15 is a measure of the neuroticism personality trait based on Eysenck's^38^ five-factor model. Developed by Wongpakaran et al., it consists of a self-rating scale including 15 items with a 0 to 4 Likert scale^39^. NI instructs that the participants match the overall appearance from their past to the present and does not mean only a certain period. A higher score reflects a higher level of neuroticism. Cronbach’s alpha was 0.83. In this study, Cronbach’s alpha was 0.905.

### Statistical analysis

The participants’ demographic data were descriptively analyzed. For numerical data, mean (SD) was used for data with normal distribution, while the median (range) was used for nonnormally distributed data. The internal consistency was calculated to evaluate the reliability of the questionnaires; Cronbach’s alpha coefficient of 0.7 or higher was considered acceptable. Pearson’s and Spearman’s rank correlation were used to examine the significant relationship between variables. Hierarchical regression analysis was performed to determine the effect of each variable on the QOL. Both EQ-5D and the impact of daily life were separately analyzed. Normal distribution tests were conducted before performing regression analysis. Kolmogorov–Smirnov and Shapiro–Wilk statistics were used for the test. The impact of daily life subscale scores yields nonsignificant results (p > 0.05), whereas the EQ-5D index value was significant, indicating that the data were nonnormally distributed. Data transformation using Log10 was applied for EQ-5D data. A P-value less than 0.05 was used to determine the significant level. SPSS Program (Version 22.0, SPSS Inc., Chicago, IL, USA) was used for data analysis.

### Ethics approval and consent to participate

This study was approved from the Research and Ethics Committee of Faculty of Medicine, Chiang Mai University (Study code: 261/2018 and date of approval, 18 July 2018) and followed the tenets of Declaration of Helsinki. Written informed consent was obtained from all the participants after complete explanation.

## Results

### Participants

Among 100 participants with DED, 89% were females with a mean age of 50.9 ± 14.4 (20–84) years. The participants’ demographic data are demonstrated in Table 1.

**Table 1:** Demography and characteristics of participants with DED (N = 100).

Characteristic	N (%)
**Age**	
Mean ± SD	50.9 ± 14.36
Range	20–84
Sex: female	89 (89.0%)
**Ocular diseases**	**80 (80.0%)**
Dry eye	75 (75%)
Cataract	11 (11%)
Pterygium	10 (10%)
Pinguecula	2 (2%)
Glaucoma	1 (1%)
**Systemic diseases**	**65 (65.0%)**
Hypertension	23 (23%)
Dyslipidemia	18 (18%)
Allergy	16 (16%)
Systemic lupus erythematosus	8 (8%)
Diabetes mellitus	5 (5%)
Inactive cancer	5 (5%)
Miscellaneous*	14 (14%)
Regular exercise	42 (42%)
Contact lens wear	4 (3.8%)
Smoking	2 (2.0%)
**DEQS-TH scores: mean (SD)**	
Summary score	43.7 ± 19.8

*DED* dry eye disease, *SD* standard deviation, *DEQS* Dry Eye-related Quality-of-Life Score, *Hypothyroidism (4), gout (2), osteoporosis (2), chronic kidney disease (1), polycystic ovarian syndrome (1), migraine (1), anemia (1), coronary artery disease (1), Gastroesophageal reflux disease (1).

Table 2 shows the mean and standard deviation of the variables, the Cronbach’s alpha values, and the significant relationship between the impact of daily life score and EQ-5D score and PSS-10 and NI-15 scores (all p < 0.01). The DED-specific QOL questionnaire appeared to moderately correlate with general health-related QOL questionnaire results measured by EQ-5D (p < 0.01).

**Table 2:** Mean score of each item of the DEQS-TH and the correlations with age, sex, NI and PSS scores using Pearson’s among participants with DED.

	Mean ± SD	Cronbach	1	2	3	4	5	6	7
1. Sex, female	–	–	–	0.096	0.061	− 0.054	0.044	0.109	0.091
2. Age	50.91 ± 14.36	–		–	− 0.023	− 0.205*	0.022	0.138	− 0.039
3. NI score	31.67 ± 9.10	0.905			–	0.472**	0.317**	0.415**	0.480**
4. PSS score	18.53 ± 6.09	0.803				–	0.320**	0.402**	0.437**
5. Eye symptoms	18.46 ± 6.10	0.901					–	0.650**	0.350**
6. Impact on Daily Life	45.44 ± 22.01	0.954						–	0.499**
7. EQ_5D index value	0.86 (0.22)^†^	0.740							–

*DEQS* Dry Eye-related Quality-of-Life Score, *SD* standard deviation, *DED* dry eye disease, *NI* Neuroticism Inventory, *PSS* Perceived Stress Scale, *EQ-5D* EuroQoL-5 dimension, *p < 0.05, **p < 0.01, ***p < 0.001, ^†^median (interquartile range).

Table 3 presents the results of the hierarchical regression model. Age was included in step 1 as a covariate and accounted for 1.8% (DEQS) and 3.9% (EQ-5D) of the variance in QOL. The score of ocular symptoms of DEQS (DEQS-Ocular symptoms) were introduced in step 2 and were a significant predictor of QOL scores, even after controlling for the covariates in step 1—explaining an additional 30.8% (DEQS) and 12.8% (EQ-5D) of the variance. Perceived stress was introduced in step 3, explaining an additional 5.2% and 13.3% of the variance and reducing the regression coefficient for the DESQ-Ocular symptoms from Beta 2.073 to 1.823 (DEQS) and 0.024 to 0.018 (EQ-5D), supporting that perceived stress mediates the relationship between DESQ-Ocular symptoms and DESQ-QOL scores. Finally, neuroticism was introduced in step 4, explaining an additional 2.7% and 6.9% of the variance and reducing the regression coefficient for both DESQ-Ocular symptoms to Beta 1.678 (DEQS) and 0.014 (EQ-5D), and perceived stress to 0.582 (DEQS) and 0.015 (ED-5D), supporting that perceived stress mediates the relationship between DESQ-Ocular symptoms and DESQ-QOL scores.

**Table 3:** Hierarchical multiple regression analyses predicting QOL score of DEQS-Th and EQ-5D from DEQS-Ocular symptoms, perceived stress, and neuroticism scores.

Step	Variable	Impact on daily life			EQ-5D		
		R^2^ adj	R^2^ change	B (95% CI)	R^2^ adj	R^2^ change	B (95% CI)
1		0.018	0.028		0.039	0.002	
	Age			− 0.258 (− 0.561, 0.045)			− 0.001 (− 0.006, 0.004)
2		0.323	0.308***		0.130	0.128**	
	Age			− 0.029 (− 0.289, 0.231)			0.002 (− 0.003, 0.007)
	DESQ-Ocular Symptoms			2.073*** (1.46, 2.686)			0.024** (0.001, 0.010)
3		0.370	0.052**		0.236	0.133***	
	Age			0.020 (− 0.234, 0.273)			0.004 (− 0.001, 0.008)
	DESQ-Ocular Symptoms			1.823*** (1.207, 2.440)			0.018*** (0.005, 0.030)
	PSS-10			0.878** (0.268, 1.488)			0.023** (− 0.017, 0.007)
4		0.391	0.027*		0.300	0.069*	
	Age			− 0.015 (− 0.267, 0.236)			0.002 (− 0.002, 0.001)
	DESQ-Ocular Symptoms			1.678*** (1.056, 2.300)			0.014* (0.002, 0.026)
	PSS-10			0.582 (− 0.081, 1.244)			0.015* (0.002, 0.028)
	NI-15			0.461* (0.022, 0.901)			0.012** (0.004, 0.020)

*QOL* quality of life, *DEQS-Th* Thai version of Dry Eye-related Quality-of-Life Score, *EQ-5D* EuroQoL-5 dimension, *adj* adjusted, *R* coefficient r, *R*^*2*^* adj* adjusted R square, *B* unstandardized Coefficients, *PSS* Perceived Stress Scale, *NI* Neuroticism Inventory, *p < 0.05, **p < 0.01, ***p < 0.001.

## Discussion

Our findings demonstrated a significant relationship between perceived stress and neuroticism and QOL regardless of measurement used, DED specific or general health related. Although DED-specific QOL was the greater contribution from dry eye symptoms as it constitutes disease-specific QOL, dry eye symptoms also demonstrated a significant relationship to general health-related QOL. This highlights the importance of dry eye symptoms on QOL^12,40^.

Even though the DEQS is designed to capture QOL related to dry eye symptoms, our results demonstrated that only 32% of the variance of the QOL explained the dry eye symptoms. Adding perceived stress and neuroticism, the variance explained increases to 39%. Dry eye symptoms explained 13% of the variance of EQ-5D and increased to 30% when combined with perceived stress and neuroticism. The reason psychological factors have a stronger effect size on EQ-5D than DEQS is that the former is closely related to anxiety/depression than the latter. In DEQS, individuals report more on the impact on function rather than anxiety/ depression, and vice versa for EQ-5D. That explains the effect that perceived stress persists along with neuroticism. However, both measurements have provided robust evidence for the role of neuroticism among patients with DED.

Dry eyes and another chronic medical disease are related to neuroticism^30,41^. Individuals with neuroticism are usually susceptible to stress and may complain disproportionally to the physician to what has been shown in the objective test^42^. Investigators have supported the relationship between subjective complaints and feelings of stress and neuroticism^28^. In addition to perceived stress, neuroticism is closely related to anxiety and depression^43–45^, that constitute high risk for worsening dry eye symptoms and poor QOL^5^, but not included in this analysis.

Most studies have endorsed the significant correlation between neuroticism and dry eye symptoms, except for one conducted by Kaiser et al.^31^. In their study, the Munich Personality Test was used to measure the personality trait, whereas most used the Big Five Personality Inventory. The different measurements might have contributed to the ability to detect differences among subjects.

As individuals with high neuroticism are emotionally reactive and exhibit a tendency to react to events that would not impact most people, symptoms disproportionately complained among patients with DED could be explained by the patient’s neuroticism. Neuroticism may be considered the central construct linking the tendency to present perceived stress, anxiety sensitivity, anxiety, and depression, that eventually affect the QOL than that obviously and objectively present by the clinical lab^24–26,28^. Screening for perceived stress and neuroticism in routine practice may help the clinician to have a better explanation and practical plan for these patient groups.

## Strength and limitations

This was the first to study neuroticism and perceived stress on DED that would allow clinicians and researchers to be aware psychosocial influence among these patients.

As the data were collected in the ophthalmology clinic, all participants completed the questionnaires in the private area before or after seeing ophthalmologists. Whether or not doing questionnaires during such a period would impact their response to each questionnaire is difficult to conclude. They were given sufficient time and privacy to ensure that all the questionnaires were completed in the same period of a given time. Psychometric property including convergent and construct validity of the measurements were not tested among this sample. Another limitation is that most participants were female. As neuroticism is dominant among females, a replication of the study among male patients is warranted.

## Conclusions

In summary, we found a significant association between perceived stress, neuroticism and the QOL of patients with DED. Our current findings suggest that the personality of the patient, which is the fundamental psychological factor has some impact on both subjective dry eye symptoms and impact on daily life, along with the general health related QOL.

## Data Availability

The datasets used and/or analyzed during the current study available from the corresponding author on reasonable request.

## Abbreviations

DED: Dry eye disease
DEQS: The Dry Eye-Related Quality of Life Score questionnaire
DEQS-TH: The Thai version of the Dry Eye-Related Quality of Life Score questionnaire
EQ-5D-5L: EuroQol‑5‑Dimensions 5‑Level
NI: Neuroticism inventory
PSS: Perceived stress scale
QOL: Quality of life

## References

[1] Stapleton F (2017). TFOS DEWS II Epidemiology Report. Ocul. Surf..

[2] Uchino M, Schaumberg DA (2013). Dry eye disease: Impact on quality of life and vision. Curr. Ophthalmol. Rep..

[3] Grubbs JR, Tolleson-Rinehart S, Huynh K, Davis RM (2014). A review of quality of life measures in dry eye questionnaires. Cornea.

[4] Barabino S, Labetoulle M, Rolando M, Messmer EM (2016). Understanding symptoms and quality of life in patients with dry eye syndrome. Ocul. Surf..

[5] Na KS, Han K, Park YG, Na C, Joo CK (2015). Depression, stress, quality of life, and dry eye disease in Korean women: A population-based study. Cornea.

[6] Li M, Gong L, Chapin WJ, Zhu M (2012). Assessment of vision-related quality of life in dry eye patients. Investig. Ophthalmol. Vis. Sci..

[7] Craig JP (2017). TFOS DEWS II Definition and Classification Report. Ocul. Surf..

[8] Nichols KK (2006). Patient-reported symptoms in dry dye disease. Ocul. Surf..

[9] Sullivan BD (2014). Correlations between commonly used objective signs and symptoms for the diagnosis of dry eye disease: Clinical implications. Acta Ophthalmol..

[10] Yu J, Asche CV, Fairchild CJ (2011). The economic burden of dry eye disease in the United States: A decision tree analysis. Cornea.

[11] McDonald M, Patel DA, Keith MS, Snedecor SJ (2016). Economic and humanistic burden of dry eye disease in Europe, North America, and Asia: A systematic literature review. Ocul. Surf..

[12] Gomes JAP, Santo RM (2019). The impact of dry eye disease treatment on patient satisfaction and quality of life: A review. Ocul. Surf..

[13] Um SB (2018). Association between dry eye symptoms and suicidal ideation in a Korean adult population. PLoS One.

[14] Al-Dairi W, Al Sowayigh OM, Alkulaib NS, Alsaad A (2020). The relationship of dry eye disease with depression in Saudi Arabia: A cross-sectional study. Cureus.

[15] Hyon JY, Yang HK, Han SB (2019). Association between Dry Eye Disease and Psychological Stress among Paramedical Workers in Korea. Sci. Rep..

[16] Wang MT, Muntz A, Wolffsohn JS, Craig JP (2021). Association between dry eye disease, self-perceived health status, and self-reported psychological stress burden. Clin. Exp. Optom..

[17] Reiss S, Peterson RA, Gursky DM, McNally RJ (1986). Anxiety sensitivity, anxiety frequency and the prediction of fearfulness. Behav. Res. Ther..

[18] Toth M, Jokić-Begić N (2020). Psychological contribution to understanding the nature of dry eye disease: A cross-sectional study of anxiety sensitivity and dry eyes. Health Psychol. Behav. Med..

[19] Telch MJ, Shermis MD, Lucas JA (1989). Anxiety sensitivity: Unitary personality trait or domain-specific appraisals?. J. Anxiety Disord..

[20] Banjongrewadee M (2020). The role of perceived stress and cognitive function on the relationship between neuroticism and depression among the elderly: A structural equation model approach. BMC Psychiatry.

[21] Gramstad TO, Gjestad R, Haver B (2013). Personality traits predict job stress, depression and anxiety among junior physicians. BMC Med. Educ..

[22] Hazlett-Stevens H, Craske MG, Mayer EA, Chang L, Naliboff BD (2003). Prevalence of irritable bowel syndrome among university students: The roles of worry, neuroticism, anxiety sensitivity and visceral anxiety. J. Psychosom. Res..

[23] Karaaslan Ö, Kantekin Y, Hacımusalar Y, Dağıstan H (2020). Anxiety sensitivities, anxiety and depression levels, and personality traits of patients with chronic subjective tinnitus: A case–control study. Int. J. Psychiatry Clin. Pract..

[24] Huang IC (2017). Does personality affect health-related quality of life? A systematic review. PLoS ONE.

[25] Bobić J (2012). Subjective estimation of the quality of life in relation to neuroticism. Arh Hig Rada Toksikol.

[26] Calkins AW, Hearon BA, Capozzoli MC, Otto MW (2013). Psychosocial predictors of sleep dysfunction: The role of anxiety sensitivity, dysfunctional beliefs, and neuroticism. Behav. Sleep Med..

[27] McCrae RR, Costa PT (1987). Validation of the five-factor model of personality across instruments and observers. J. Pers. Soc. Psychol..

[28] Ichinohe S, Igarashi T, Nakajima D, Ono M, Takahashi H (2016). Symptoms of dry eye disease and personality traits. PLoS One.

[29] Feroze KB, AlAbdullah ZAM, AlOnayzan AHA, Pattath A (2020). The association between personality traits and dry eye disease: A cross-sectional study. Saudi J. Ophthalmol..

[30] Milic V (2019). Personality, depression and anxiety in primary Sjogren's syndrome—Association with sociodemographic factors and comorbidity. PLoS One.

[31] Kaiser T, Janssen B, Schrader S, Geerling G (2019). Depressive symptoms, resilience, and personality traits in dry eye disease. Graefes Arch. Clin. Exp. Ophthalmol..

[32] Wolffsohn JS (2017). TFOS DEWS II Diagnostic Methodology report. Ocul. Surf..

[33] van Bijsterveld OP (1969). Diagnostic tests in the Sicca syndrome. Arch. Ophthalmol..

[34] The EuroQol Group (1990). EuroQol—A new facility for the measurement of health-related quality of life. Health Policy.

[35] Tongsiri S, Cairns J (2011). Estimating population-based values for EQ-5D health states in Thailand. Value Health.

[36] Cohen S, Kamarck T, Mermelstein R (1983). A global measure of perceived stress. J. Health Soc. Behav..

[37] Wongpakaran N, Wongpakaran T (2010). The Thai version of the PSS-10: An investigation of its psychometric properties. Biopsychosoc. Med..

[38] Eysenck HJ, Eysenck SBG (1975). Manual of the Eysenck Personality Questionnaire.

[39] Wongpakaran N (2019). Prevalence, clinical and psychosocial variables of depression, anxiety and suicidality in geriatric tertiary care settings. Asian J. Psychiatry.

[40] Dana R, Meunier J, Markowitz JT, Joseph C, Siffel C (2020). Patient-reported burden of dry eye disease in the United States: Results of an online cross-sectional survey. Am. J. Ophthalmol..

[41] Sutin AR, Zonderman AB, Ferrucci L, Terracciano A (2013). Personality traits and chronic disease: Implications for adult personality development. J. Gerontol. B Psychol. Sci. Soc. Sci..

[42] Zijlema WL, Morley DW, Stolk RP, Rosmalen JG (2015). Noise and somatic symptoms: A role for personality traits?. Int. J. Hyg. Environ. Health.

[43] Kim SE (2016). Direct and indirect effects of five factor personality and gender on depressive symptoms mediated by perceived stress. PLoS One.

[44] Pereira-Morales, A., Adan, A. & Forero, D. *Perceived Stress as a Mediator of the Relationship Between Neuroticism and Depression and Anxiety Symptoms* (2017).

[45] Suradom C (2019). Prevalence and associated factors of comorbid anxiety disorders in late-life depression: Findings from geriatric tertiary outpatient settings. Neuropsychiatr. Dis. Treat..

